# The Management of Children and Adolescents with Overactive Bladder Refractory to Treatment with Parasacral Transcutaneous Electrical Nerve Stimulation

**DOI:** 10.1590/S1677-5538.IBJU.2024.0453

**Published:** 2025-01-10

**Authors:** Carlos Eduardo Rocha Macedo, Antônio Vitor Nascimento Martinelli Braga, Felipe Santos Marimpietri, Beatriz Paixão Argollo, Glicia Estevam de Abreu, Maria Luiza Veiga da Fonseca, Ana Aparecida Nascimento Martinelli Braga, Ubirajara Barroso

**Affiliations:** 1 Escola Bahiana de Medicina e Saúde Pública Centro de Distúrbios Urinários Infantis Salvador Bahia Brasil Centro de Distúrbios Urinários Infantis (CEDIMI), Escola Bahiana de Medicina e Saúde Pública, Salvador, Bahia, Brasil

**Keywords:** Child, Urinary Bladder, Overactive, therapy [Subheading]

## Abstract

**Background:**

Although parasacral TENS (pTENS) has been employed in various centers, there is a lack of studies on how children with overactive bladder (OAB) respond after failing to complete pTENS sessions. This study aimed to describe and assess treatments for OAB in children who did not respond to pTENS.

**Material and Methods:**

This retrospective case series examined patients aged 4-17 years. Patients were given subsequent treatment options, including: behavioral therapies; oxybutynin; imipramine; a combination of oxybutynin and imipramine; parasacral percutaneous electrical nerve stimulation (PENS); or a repeat course of pTENS. Outcomes were evaluated using the Dysfunctional Voiding Scoring System (DVSS) and the Visual Analogue Scale (VAS).

**Results:**

Thirty children were included, with a median age of 7 years. Patients received one or more treatments. Of these, 70% underwent monotherapy. Among them, 57% experienced complete resolution of symptoms, 28% had partial resolution and were satisfied with the results, and 14% discontinued treatment. 30% out of the whole sample continued to experience bothersome symptoms. Complete response, according to initial subsequent, was achieved in: 54% with intensified behavioral therapies, 33% with oxybutynin, and 50% with imipramine alone. The median DVSS score decreased from 7.0 to 2.0 (p=0.025), while the median VAS score increased from 80 to 100 (p<0.001).

**Conclusion:**

Children with OAB refractory to pTENS who received structured subsequent treatments showed partial response in all cases, with complete symptom resolution in half of the patients. More intensive urotherapy, medications, or repeat pTENS in combination with oxybutinin can be effective for managing this challenging condition.

## INTRODUCTION

Lower urinary tract dysfunction (LUTD) is a disorder resulting from abnormalities in the filling or voiding phases of the bladder in individuals with no associated neurological abnormality. Overactive bladder (OAB) is the most prevalent form of LUTD, and, from a clinical point of view, it manifests as the presence of urgency to void, associated or not with incontinence, nocturia or pollakiuria (1). Due to the impact of urinary symptoms in the patient´s quality of life and its association with vesicoureteral reflux and recurrent urinary tract infections, the timely management of OAB is essential (2, 3). According to ICCS, voiding diary is one of the three diagnosis tests included in the non-invasive urodynamic (1). Franck HHM et al. (2023) have shown that only one voiding diary is enough to assess these children (3).

Behavioral therapies involving lifestyle changes have been used as first-line therapy for OAB. However, a certain proportion of patients requires further assistance (4). The pharmacological treatment using oxybutynin represents an efficient treatment option, with an improvement rate of 60% and a complete resolution rate of 30%. Despite benefits, oxybutynin can trigger undesired side effects, such as dry mouth, constipation, and other symptoms that lead to a drop rate of 10% (5). Bladder wall thickness measurements and nerve growth factor (NGF) / creatinine values could predict the outcomes in this population (6). Furthermore, vitamin D deficiency is more common in children with urinary incontinence and OAB than in healthy children. Vitamin D supplementation may improve urinary symptoms and quality of life in patients with OAB (7).

In this respect, parasacral transcutaneous electrical nerve stimulation (parasacral TENS) has been used as an option for the treatment of OAB. This treatment modality consists of a non-invasive technique with no significant side effects that also has an additional beneficial effect on constipation (5). The rate of complete resolution of urinary symptoms with TENS is similar to that achieved with pharmacological treatment (3). However, in around 40% of patients who undergo treatment with TENS, symptoms will not resolve completely (8).

Although various studies have dealt with the treatment of this dysfunction, to the best of our knowledge, up to the moment no studies in the literature have described and analyzed the therapeutic management of patients who fail to respond to parasacral TENS. Such studies are necessary to support decision-making in the case of patients with OAB refractory to parasacral TENS. We hypothesize that children and adolescents with OAB who failed to respond to parasacral TENS can still achieve complete resolution of symptoms after further assistance. Therefore, the objective of the present study was to describe and evaluate the treatments given to patients with OAB who failed to respond to parasacral TENS.

## MATERIAL AND METHODS

### Study design

This is a descriptive, longitudinal, analytical case series conducted by evaluating the records of patients undergoing treatment at a specialist center. The institution's internal review board approved the study protocol under reference number / CAAE: 13212419.3.0000.5544.

### Sample selection

The inclusion criteria were patients of 4-17 years of age with non-complicated OAB refractory to two or three 20- minute sessions a week of parasacral TENS, for a total of 20 sessions. Patients no longer being followed up at the center were contacted by telephone to obtain the relevant data.

All patients included in the present study have failed standard urotherapy for the period of one month before being submitted to parasacral TENS. No patients included had any upper tract involvement or major complications.

Children and adolescents whose records were incomplete in relation to the principal endpoint and with neurodevelopment or psychiatric issues were excluded from the study.

Electrical nerve stimulation was performed using a Dualpex Quark 961 device (Quark Medical, Piracicaba, Brazil) at a frequency of 10HZ, pulse width of 700 microseconds, over two or three 20-minute sessions a week for a total of 20 sessions.

### Evaluation instruments

The data collected included: Age; Gender; The different types of treatments and associations instituted during follow-up; Scores obtained using the Dysfunctional Voiding Scoring System (DVSS) and the visual analogue scale (VAS), applied to evaluate symptoms prior to and following treatment. The version of the DVSS validated for use in the Portuguese language was used to determine the severity of symptoms, with scores ≥ 6 for girls and 9 for boys being indicative of severe LUTD and a post-treatment score of 0 being considered complete resolution of symptoms [6]. The VAS is used to evaluate clinical improvement, with scores ranging from 0 to 10 in which 0 reflects no improvement at all and 10 is indicative of complete resolution [7]. These VAS scores were then multiplied by a factor of 10 to calculate the percentage of improvement. The center routinely collects these data for all patient visits.

Patients who failed to respond to TENS were given a subsequent treatment by using one of the following treatment options: Behavioral therapies; Oxybutynin; Imipramine; Oxybutynin in combination with imipramine; Parasacral percutaneous electrical nerve stimulation (PENS) or a repeat course of parasacral TENS.

The following criteria were used to select treatment after parasacral TENS has failed.

Patients with few symptoms, particularly those in whom urinary urgency was associated with voiding postponement, were instructed to continue with and intensify behavioral therapy.

For more symptomatic patients, oxybutynin was prescribed, and the dose of the drug was increased until adequate therapeutic response was obtained or adverse events developed. If side effects occurred or the patient failed to respond to oxybutynin, imipramine was used.

When the patient failed to respond to oxybutynin or imipramine alone, the two drugs were then given in combination.

When parents chose not to have their child undergo pharmacological treatment since the drug would have had to be used over a period of some months, preferring to repeat parasacral TENS or try a new type of treatment, parasacral PENS [8].

All patients with psychological issues were managed by our team of psychologists.

All constipated patients had the bowel dysfunction treated by our proctologist.

Due to our patients’ inability to afford mirabegron, we did not test the use of this medication. Patients were reassessed every 3 months to analyze treatment outcomes, regardless of the treatment modality.

### Statistical analysis

The data collected were stored and analyzed using the SPSS software program, version 14.0. The results are presented as graphs. The dependent variables were the treatments given after TENS had failed, while the independent variables were: Sex; Age; DVSS score and VAS score. The numerical data from the DVSS and VAS were expressed as medians and interquartile ranges (IQR). The categorical variables (the treatments instituted and the number of patients who went on to achieve complete resolution of symptoms) were expressed as frequencies (%). To compare the median DVSS and VAS scores prior to and following each type of treatment, the Wilcoxon test was used after the normality of distribution had been determined. P-values <0.05 were considered statistically significant.

## RESULTS

Thirty children, 20 of whom were girls, with a median age of 7.0 years (IQR: 5-10 years) who have failed to respond to parasacral TENS were included in the study. Failure to respond to TENS consisted of the persistence of symptoms in 29 cases (97%) and symptom recurrence after an initial period of complete resolution in one case (3%). Median follow-up time was 9 months (IQR: 3.25- 26 months).

### Daytime symptoms before the subsequent treatment

All the patients continued to have daytime symptoms. Some patients had more than one symptom, with 6 (20%) having three associated symptoms and 11 (36%) having only two associated symptoms. Table-1 shows demographic information of the sample, frequency of symptoms before the subsequent treatments and median VAS score before and after the subsequent treatments, according to the gender.

**Table 1:** Demographic data, symptom frequency before the subsequent treatments and median VAS scores before and after the subsequent treatments, according to the gender.

Variables		Male (N=10)	Female (N=20)	Total (N=30)
Median Age (IQR)		5 (4.75-7.25)	7.5 (6-10)	7 (5-10)
**Symptoms (%)**				
	Urinary urgency	8 (80%)	11 (55%)	19 (63%)
	Urge-incontinece	6 (60%)	13 (65%)	19 (63%)
	Nocturnal enuresis	8 (80%)	10 (50%)	18 (60%)
	Incontinence without urgency	3 (30%)	5 (25%)	8 (26%)
	Holding maneuvers	3 (30%)	4 (20%)	7 (23%)
	Pollakiura	0 (0%)	2 (10%)	2 (6%)
**Median VAS scores (IQR)**				
	Before subsequent treatments	65 (50-90)	80 (50-87,5)	80 (50-90)
	After subsequent treatments	100 (67,50-100)	90 (82,50-100)	100 (80-100)

IQR: Interquantil Range; VAS: Visual Analogue Scale

The median VAS score was 80 (IQR: 50-90). In one case, in which symptoms recurred after having been completely resolved, the VAS score was 100%, indicating complete resolution at that moment.

### Outcome after subsequent treatments

**Figure 1:**
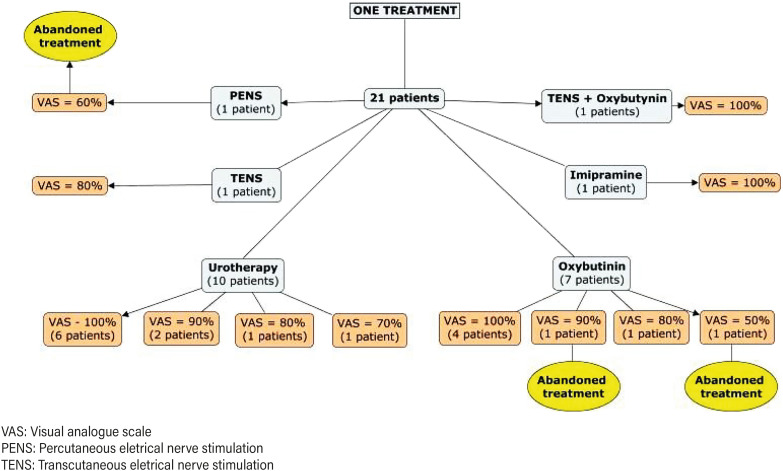
Treatment given, its efficacy according to VAS score and the cases of abandonment treatment for patients who underwent only one treatment.

**Figure 2:**
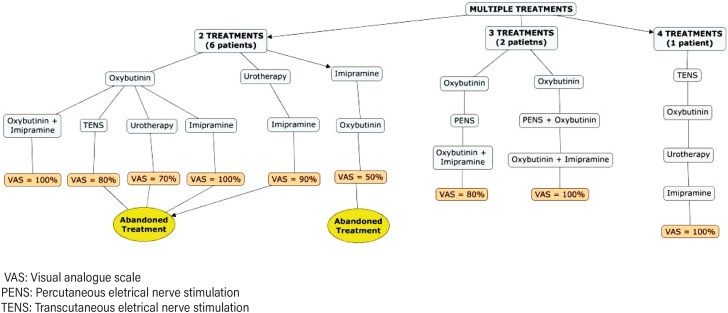
Sequence in which treatments were given, efficacy of the treatment according to the VAS score, and the cases of abandonment treatment for patients who underwent multiple treatments.

During the follow-up period, after the patients failed to parasacral TENS, they were then submitted to one or more types of treatment. A total of 21 patients (70%) underwent monotherapy (Figure-1). Of them, 12 patients (57%) had complete resolution of the symptom, 6 (28%) had partial resolution and were satisfied with the results and 3 (14%) abandoned treatment. Nine patients out of the whole sample (30%) maintained bothersome symptoms and required further assistance after the first subsequent treatment has failed. For these patients, we offered different or adjuvant therapies (Figure-2).

The median DVSS score decreased from 7.0 (IQR: 3-9) prior to the first treatment instituted post- TENS to 2.0 (IQR: 0.2-6.5) after treatment (p=0.025). The median VAS score increased from 80 (IQR: 50-90) to 100 (IQR: 80-100), respectively (p<0.001). According to the DVSS score, complete resolution of symptoms was achieved in 11 patients (36%), 9 of whom were girls., symptoms were completely resolved in 16 patients (53%) (Table-2).

**Table 2:** The VAS scores before subsequent treatments (immediately following parasacral TENS) and the VAS scores after the other treatments provided.

VAS scores	Before subsequent treatments (N=30)	After subsequent treatments (N=30)
**0**	2 (6%)	0 (0%)
**40**	2 (6%)	0 (0%)
**50**	6 (20%)	0 (0%)
**60**	5 (16%)	2 (6%)
**70**	4 (13%)	0 (0%)
**80**	4 (13%)	4 (13%)
**90**	2 (6%)	6 (20%)
**100**	5 (16%)	18 (60%)

VAS: Visual analogue scale

According to the success rate of initial subsequent treatment modality, complete response to symptoms was achieved in 6/11 patients (54%) who underwent intensified behavioral therapies, in 4/12 (33%) who used oxybutynin, in 1/2 (50%) who used imipramine alone and in the only patient submitted to further sessions of parasacral TENS associated with oxybutynin. The only patient submitted to another series of TENS alone as initial subsequent treatment modality did not achieve complete response to symptoms.

For the patients who failed to respond to the initial subsequent treatment modality, different therapies were given. Complete resolution of symptoms was achieved in 1 out of the 2 patients (50%) who continued treatment with oxybutynin, in 1/2 patient (50%) who were submitted to continued urotherapy, in 2/3 (66%) patients who used oxybutynin associated with imipramine and in 2/3 (66%) patient who used imipramine alone.

## DISCUSSION

Our study demonstrated that after TENS failure, 53% patients with LUTD had complete response to subsequent treatment and all achieved at least partial response of the symptoms. Our data shows that 30% need further treatment, demonstrating a multimodal and multiprofessional approach is needed for treatment of the LUTD. This is the first study that describes the outcome of patients who underwent further treatment after parasacral TENS fails for this condition.

Behavioral therapies (urotherapy) are the first-line treatment for children with OAB who have never been treated before (3). In this study, symptoms were completely resolved in 6 of the 11 patients who continued with the urotherapy as the first treatment following parasacral TENS. It is important to underline that standard urotherapy was maintained and reinforced in cases of children with holding maneuvers who had failed to follow the instructions correctly. These data highlight the difficulty that parents and their children go through to maintain this treatment over the long- term, but reinforce the importance of persisting with standard urotherapy. In this respect, the work of a multidisciplinary team may help in assuring the compliance of the children and their families with the treatment.

Oxybutynin is widely used as second-line therapy for children with OAB, with an efficacy rate of 30-40% (3, 11). In the present study, oxybutynin was the most used option, particularly for patients with persistency of overactive bladder symptoms. When oxybutynin was used alone as first line treatment after parasacral TENS failure, 50% of the patients had their symptoms completely resolved. It is surprisingly that we did not find in the literature any study of oxybutynin for refractory patients.

In a randomized clinical trial, investigators from Aarhus, Denmark, showed that the combination of oxybutynin with parasacral TENS was more effective than either treatment alone (12). If this association had been used more frequently in the present study, it is possible that the results could have been better. However, the associations of oxybutynin with electrical nerve stimulation (PENS + oxybutynin and TENS + oxybutynin) were used in too few patients. In short, one patient underwent treatment with TENS + oxybutynin, resulting in complete resolution of symptoms, and one patient was treated with PENS + oxybutynin, resulting in a reduction in the DVSS score but no improvement in the VAS score.

We evaluated 2 patients who underwent another series of electrical nerve stimulation. One patient, refractory to TENS was treated with PENS, a more invasive type of stimulation, had a final VAS score of 60 and did not come back to the clinic for follow-up. Other patient who had recurrency of the urinary symptoms after an initial complete resolution of them, underwent a new series of parasacral TENS, achieving a VAS score of 80 and showing that, for these cases, redo TENS can be a good option.

Imipramine has been shown beneficial for some children with OAB who have failed to respond to other forms of treatment (11); however, it is still not considered a standard treatment due to the lack of data from randomized clinical trials. In the study conducted by Franco et al. (12), symptoms were resolved in 42.7% of the patients with refractory LUTD who went on to use imipramine, while 22.3% had a partial improvement, 15.5% obtained no response to treatment and 19.5% were lost to follow-up. In the present series, complete resolution of symptoms was achieved in 1 out of 2 patients treated with imipramine as the first subsequent treatment after parasacral TENS failure and in 2 out of 3 patients who used it as alternative for further treatment, suggesting that this is a viable option for cases refractory to TENS.

The combination of oxybutynin with imipramine tends to be used in more severe cases; however, no studies have yet been conducted to evaluate the efficacy of this combined treatment on OAB. The few studies that have been published refer to children with monosymptomatic enuresis, with 66.6% of patients obtaining complete resolution of symptoms (13). In the present study, this treatment option was used primarily for patients who had failed to respond to other forms of treatment. Complete resolution of symptoms was achieved in 2 out of 3 patients who used this strategy as an alternative therapy to other treatments, suggesting that this could be a good option for more refractory cases.

There are some limitations associated with the present study, including the small number of patients and its retrospective nature and the fact that some patients were lost prior to completing treatment. Treatment abandonment may have occurred due to one of two reasons: 1) Patients were sufficiently satisfied with the results achieved even before symptoms had completely disappeared; or 2) Patients became demotivated due to the slowness of progress in extremely refractory cases. Therefore, since many patients abandoned treatment, it was impossible to evaluate the progress made in the whole sample until complete resolution of symptoms.

## CONCLUSIONS

For children with OAB refractory to TENS who underwent a structure subsequent treatment, partial response was achieved in all patients and the symptoms completely resolved in half the patients undergoing further treatment. A more intense urotherapy, medications or redo TENS in combination with oxybutynin can be successful used for this difficult situation. For case of recurrence of symptoms after a period of complete resolution of them, redo TENS alone can be useful. It is important to remember that all patient with LUTD who fail to treatment need reevaluation of the bowel function and psychological status.
